# Case Report of Nonfamilial Cherubism in a Toddler: Description of Clinic-Radiographic Features and Osseous-Dental Treatments

**DOI:** 10.1155/2016/8795765

**Published:** 2016-12-25

**Authors:** Mitra Karbasi Kheir

**Affiliations:** Department of Oral and Maxillofacial Radiology, School of Dentistry, Islamic Azad University Isfahan, Khorasgan Branch, Isfahan, Iran

## Abstract

Cherubism is a rare familial disease that occurs between the ages two and five years and regresses after puberty. Most of the cherubism cases show familial history, but there are some cases without familial histories of disorder. A two-year-old boy with a painless symmetrical progressive swelling of the jaws had visited maxillofacial radiology department. Panoramic radiograph revealed well-defined multilocular, radiolucent areas of both jaws. Computed tomography of the jaws showed well-defined, bilateral, multilocular, expansile lesions with thinning of cortical plate of maxilla and mandible and displacing the unerupted first molar anteriorly. Clinical, radiologic, and histopathologic characteristics confirmed the diagnosis of cherubism.

## 1. Introduction

Cherubism is a rare familial disease that occurs between the ages two and five years and regresses after puberty. The disease is an autosomal dominant disorder. Cherubism is known to be related to the mutations in the gene encoding the binding protein SH3BP2 on chromosome 4p16.3. Fibro-osseous tissue replaces the normal bone, leading to bilateral jaw enlargement. It was first described in 1933. Radiographically, the lesions exhibit bilateral, multilocular, radiolucent areas that often affect both maxilla and mandible. The lesion epicenter is in posterior aspect of jaws, ramus, and tuberosity in mandible and maxilla, respectively. It grows in an anterior direction and can displace the teeth in that direction. Bilateral enlargement of mandible produces rounded face and swollen cheeks. Skin over the swelling is stretched, pulling the lower eyelids down and exposing a line of sclera. A rim of sclera may be visible beneath the iris, giving the classic “eye raised to heaven” appearance. Boys are more affected than girls with the proportion of 2 : 1. Although the condition is known to regress spontaneously at puberty, surgical management is sometimes required for cosmetic reasons [1–11].

## 2. Case Report

**Figure 1:**
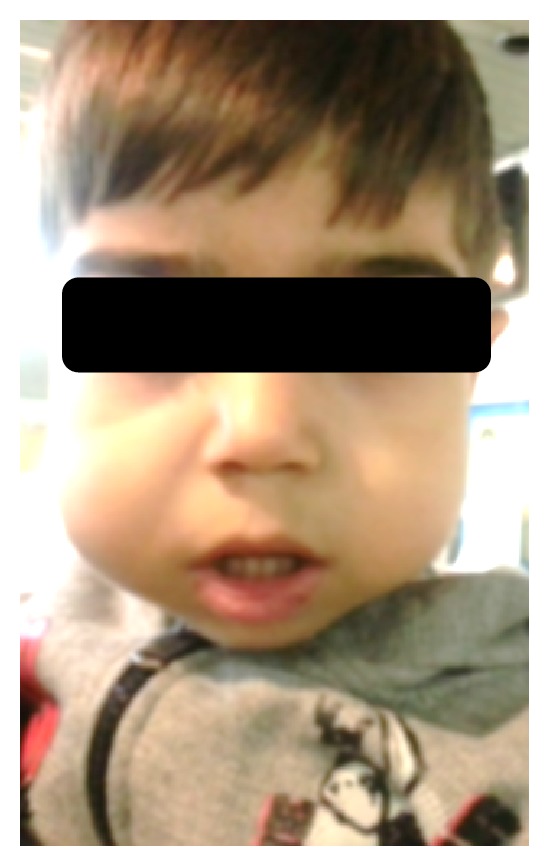
Swelling of the patient's jaws.

**Figure 2:**
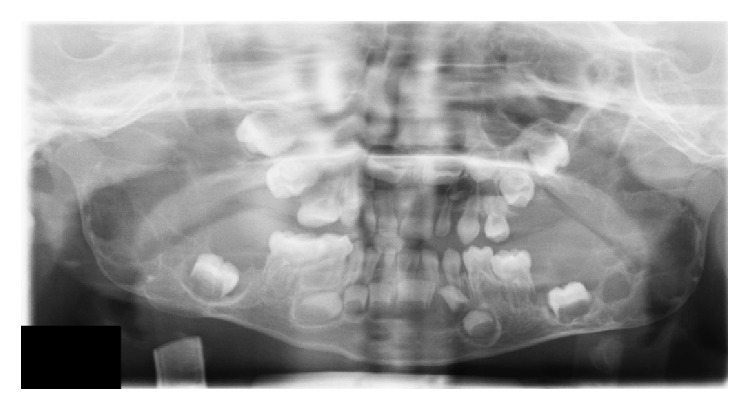
Panoramic radiograph of the lesion.

**Figure 3:**
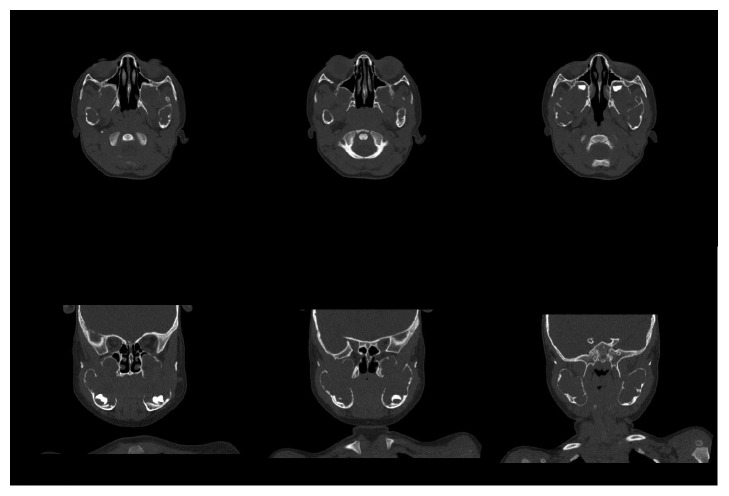
Axial and coronal views of lesion in maxilla and mandible.

**Figure 4:**
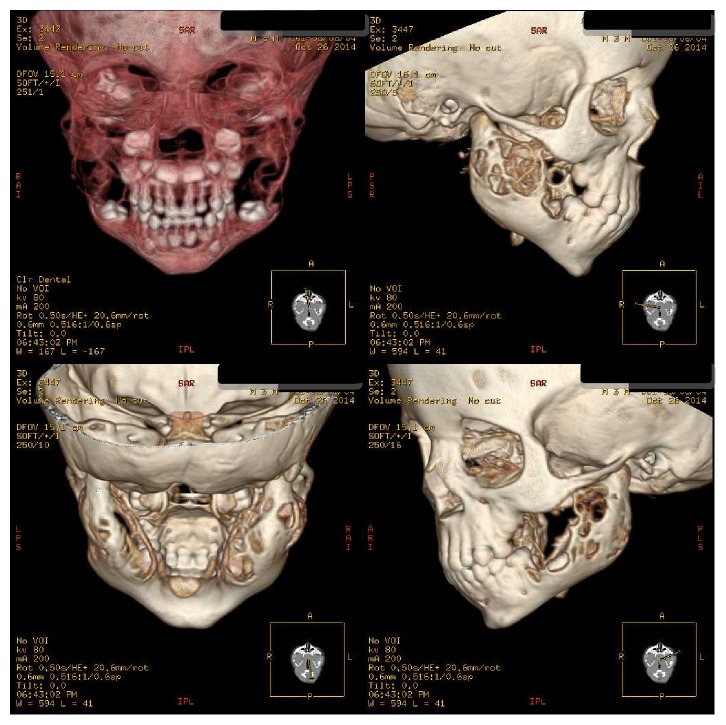
Three-dimensional images of the lesion.

**Figure 5:**
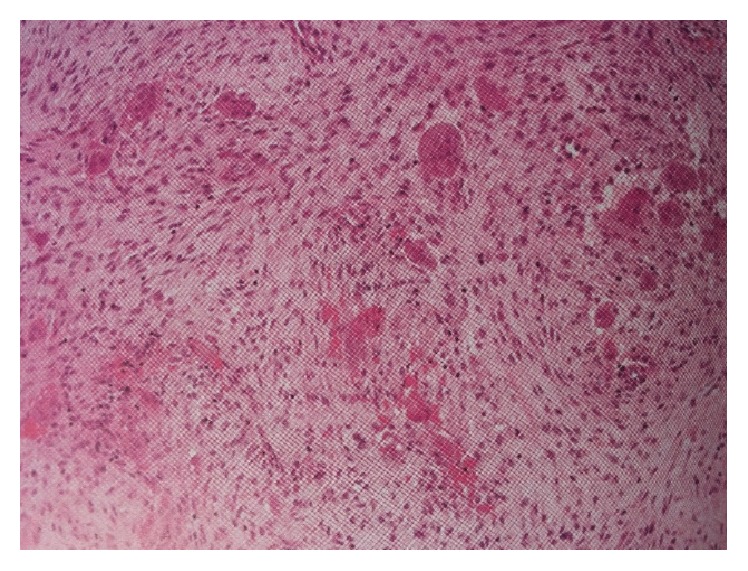
Histopathological evaluation of the lesion.

A two-year-old boy had visited maxillofacial radiology department of school of dentistry. The boy presented with a painless symmetrical progressive swelling of the jaws (Figure 1). No physical abnormalities were present. Hematological tests were normal. Intraoral clinical examination disclosed intact and blue-gray overlying mucosa. The submandibular and cervical lymph nodes were not palpable. Panoramic radiograph revealed well-defined, multilocular, radiolucent areas involving the mandibular body, angles and both ascending rami, and the maxilla (Figure 2). Computed tomography (CT) scan of the jaws showed well-defined, bilateral, multilocular, expansile lesions with thinning of cortical plate of maxilla and mandible and mild displacement of the unerupted first molar anteriorly. There were no cortical breaks, fractures, or periosteal reactions within the jaw bone and extraosseous soft tissue extension (Figures 3 and 4). Histopathological evaluation of an incisional biopsy of the lesion showed a large number of giant cells with variable size and a variable number of cores in a mesenchymal stroma with oval and fusiform cells (Figure 5). These characteristics confirm the diagnosis of cherubism. Since the disorder spontaneously regresses after puberty, there was no need to perform surgery. But the patient was advised to have regular fallow-up evaluation until puberty.

## 3. Discussion

Cherubism is a rare, inherited, autosomal dominant disease that causes the enlargement of the jaws. The lesion is bilateral and often involves both jaws. Mandible is the most common location, when only one jaw is involved. Radiographically, the internal structure of cherubism is like central giant cell granuloma with fine, granular bone, and wispy trabeculae, forming a multilocular appearance. The periphery of lesion is usually well-defined. Maxillary lesions can enlarge into maxillary sinuses. The lesion can displace the tooth in anterior direction and can sometimes destroy the dental buds or lead to abnormal patterns of teeth eruption [12]. In this case, the unerupted first molar was mildly displaced anteriorly due to the lesion. Ectopic tooth eruption, missing of posterior permanent teeth, mainly in the second and third molars, may occur in cherubism. Early exfoliation of deciduous teeth, tooth displacement, and impaction result in mal occlusion [8, 13]. In this case, the tooth buds of first and second premolars and second molar were not seen in the panoramic view. Although the lesion extended anteriorly near the second deciduous molar, considering the patient age and the probability of delay in formation and calcification of posterior tooth buds, the judgment about permanent teeth missing should be delayed and evaluation of missing teeth will be performed some years later according to the next radiographs. The patient had no caries in his teeth in radiographic and clinical evaluation, so, in this stage, no dental treatment was required. However, regular follow-up was suggested for evaluation of teeth condition. Because the lesion does not extend anteriorly to primary molar area, routine treatment (pulpectomy, extraction, etc.) for deciduous molars can be performed, if it is required in future. However, extraction of impacted molar teeth will be postponed until the lesion regression. Orthodontic treatment for teeth alignment and prosthodontic treatments for teeth replacement may be necessary in future provided that the patient misses some of his teeth due to caries and extraction. If permanent second and third molar buds are destroyed by the lesion and if the lesion results in the impaction of first permanent teeth and leads to the tooth extraction, prosthodontic replacement of teeth by partial removable dentures will be required to improve the ability to chew. Orthodontic treatment is performed after the regression of condition. Implant placement may be considered for the patient after the age of eighteen, if the lesion regresses and the quality of bone is appropriate for osseointegration.

Ramon and Engelberg described a grading system for cherubism according to the lesion extension [14] as follows:

Grade 1: involvement of both mandibular ascending rami
Grade 2: involvement of both mandibular ascending rami and maxillary tuberosities
Grade 3: massive involvement of whole maxilla and mandible, except the condylar processes
Grade 4: massive involvement of whole maxilla and mandible, except the condylar processes with involvement of the floor of the orbits causing orbital compression; according to this grading system, our patient belonged to grade 2 cherubism in which the lesion involved both mandibular ascending rami and maxillary tuberosities

Giant cell granuloma, hyperparathyroidism, Noonan's syndrome, Ramon syndrome, fibrous dysplasia, Jaffe-Campanacci syndrome, Neurofibromatosis type 1, and multiple odontogenic keratocysts are considered in differential diagnosis of cherubism. However, bilateral symmetry and posterior epicenter of cherubism can help to differentiate them. In addition, fibrous dysplasia is unilateral and does not show the swollen cheeks or upward turning of eyes, which are the characteristics of cherubism. Giant cell granuloma is usually unilateral and is mostly seen in the anterior of mandible in the age range of 20 to 40 years. It is not inherited and does not regress in adulthood. Hyperparathyroidism may be differentiated by analysis of parathyroid hormone levels, calcium, phosphorous, and alkaline phosphatase. However, hyperparathyroidism is rare in children. Noonan/multiple giant cell lesion syndrome can also be identified by genetic testing. Ramon syndrome is extremely rare with only 8 cases reported in the literature and presented with mental retardation, short stature, gingival fibromatosis, and epilepsy. Jaffe-Campanacci syndrome is a rare syndrome and includes unilateral nonossifying fibromatic lesions. The radiologic characteristic of cherubism is more diagnostic than histopathologic [1, 12, 13, 15].

This case, on the base of the family history, was probably nonfamilial because there were no other members of the family with a similar condition. Most of the cherubism cases show familial history, but there are some cases without familial histories of disorder [16, 17].

The lesion becomes static and fills in with granular bone at the end of skeletal growth. Thus, the treatment can be delayed. Indeed, waiting for disease regression is mostly recommended. After the completion of skeletal growth, conservative surgery may be required for the correction of cosmetic problems to return the contour of enlarged bone [12]. Cosmetic surgery contains simple contouring of lesion or liposuction to reduce the mass of lesion [6, 7]. However, the general policy in severe cases with functional impairments such as nasal obstruction, proptosis, speech, hearing, and swallowing problems is early surgical intervention prior to puberty. Options for surgical management include partial resection, contour resection, curettage, or a combination of these [18]. As in some cases, curettage or jaw osteoplasty has been performed to stimulate bone regeneration [8]. Nevertheless, some authors have reported rapid growth of lesion due to surgical contouring during the active phase [19]. Some literature has suggested Calcitonin therapy as an antiresorptive agent to reduce the cystic lesions of cherubism like central giant cell granuloma, but this treatment is not preferred in the rapidly growing lesions [17, 20]. Spontaneous transformation of cherubic lesions to malignancy has not been reported. However, Shah et al. reported leiomyosarcoma of mandible in a child with cherubism after two surgical recontouring procedures [21]. Of course, radiotherapy has contraindication in cherubic lesions because of the probable risk of malignancy (osteosarcoma), osteoradionecrosis, and retardation of jaw growth [17, 22].
